# Combinational antibody detection approach increases the clinical validity of colorectal cancer screening

**DOI:** 10.1002/jcla.24978

**Published:** 2023-11-14

**Authors:** Sohei Kobayashi, Takaki Hiwasa, Kouichi Kitamura, Masayuki Kano, Tyuji Hoshino, Sho Hirano, Mayuko Hashimoto, Masanori Seimiya, Hideaki Shimada, Fumio Nomura, Hisahiro Matsubara, Kazuyuki Matsushita

**Affiliations:** ^1^ Department of Laboratory Medicine & Division of Clinical Genetics Chiba University Hospital Chiba Japan; ^2^ Department of Medical Technology & Sciences, School of Health Sciences at Narita International University of Health and Welfare Chiba Japan; ^3^ Department of Neurological Surgery, Graduate School of Medicine Chiba University Chiba Japan; ^4^ Department of Frontier Surgery, Graduate School of Medicine Chiba University Chiba Japan; ^5^ Department of Physical Chemistry, Graduate School of Pharmaceutical Sciences Chiba University Chiba Japan; ^6^ Department of Gastroenterological Surgery, Graduate School of Medicine Toho University Tokyo Japan

**Keywords:** AlphaLISA, colorectal cancer, combinational antibody detection approach, FIRΔexon2 autoantibody, SEREX

## Abstract

**Background:**

At different stages of the disease, biomarkers can help to determine disease progression and recurrence and provide a personalized indicator of therapeutic effectiveness. The serological identification of antigens by recombinant cDNA expression cloning (SEREX) has identified five SEREX antigens.

**Results:**

Compared with healthy donors, anti‐FIRΔexon2 and anti‐SOHLH antibodies (Abs) in the sera of patients with colorectal cancer (CRC) were markedly higher. Furthermore, no correlation was noted between five SEREX antigens and the three tumor markers (CEA, CA19‐9, and anti‐p53 Abs), indicating that anti‐FIRΔexon2 Abs are an independent candidate marker for patients with CRC. Generally, the levels of anti‐FIRΔexon2 Abs combined with clinically available tumor markers were determined to be significantly higher compared with CEA, CA19‐9. Moreover, in early‐stage CRC, the levels of anti‐FIRΔexon2 Abs combined with existing tumor markers were higher than those of CEA, CA19‐9.

**Conclusion:**

Due to the highly heterogeneous nature of CRC, a single tumor marker is unlikely to become a standalone diagnostic test due to its commonly insufficient sensitivity and/or specificity. Using a combination antibody detection approach of tumor markers for CRC diagnosis has the potential to be an effective approach. Therefore, the use of serum protein biomarker candidates holds promise for the development of inexpensive, noninvasive, and inexpensive tests for the detection of CRC.

## Graphical abstract

The AUC values increased in the combined ROC analysis compared with the individual ROC analysis. Using a combination antibody detection approach of tumor markers for CRC diagnosis has the potential to be an effective approach. Therefore, the use of serum protein biomarkers holds promise for the development of inexpensive, noninvasive, and inexpensive tests for the detection of CRC.

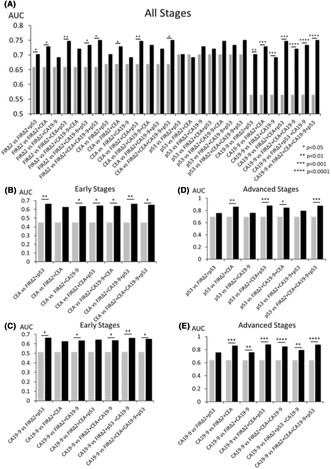

## 1 INTRODUCTION

Colorectal cancer (CRC) is the fourth most common cancer worldwide, contributing to 9.7% of the global cancer burden.^1^, ^2^, ^3^, ^4^ Declining mortality due to improvements has been shown with early detection through screening and effective treatment.^5^, ^6^, ^7^, ^8^, ^9^ The Veterans Health Administration National Center for Health Promotion and Disease Prevention recommends that men and women aged 50 to 75 years at average risk are evaluated by annual fecal immunochemical tests (FITs), sigmoidoscopy every 5 years, or colonoscopy every 10 years.^10^ However, the detection of CRC and precursor adenoma was examined in 932 patients, with a positive colonoscopy sensitivity of 60.9% and 4.7% CRC detection after FIT. CRC screening programs still have room for improvement regarding false‐positive rates for FIT and negative rates for colonoscopy.^10^ Furthermore, carcinoembryonic antigen (CEA) has proven to be beneficial in prognosis and follow‐up but has limited sensitivity (30%–40%) to detect early CRC.^11^ Therefore, further research is needed on the discovery and development of effective biomarkers for the diagnosis, prognosis, and treatment of CRC. Serological identification of antigens by recombinant cDNA expression cloning (SEREX) is an effective screening method to identify serum antibody (Ab)‐type tumor markers as multiple specific immune responses of patients with cancer.^12^ SEREX determined tumor‐related antigens as potential novel diagnostic marker candidates for types.^13^, ^14^

In our previous study, we identified five SEREX antigens in sera of patients with esophageal squamous cell carcinoma (ESCC) by the expression cloning assay through λZAP II phage library construction, including far‐upstream element‐binding protein‐interacting repressor‐lacking exon2 (FIRΔexon2; accession number: NM_001271099.1),^15^, ^16^ lysyl‐tRNA synthetase (KARS; accession number: NM_001130089.1),^17^, ^18^ sorting nexin 15 (SNX15; accession number: NM_013306.4),^19^ spermatogenesis and oogenesis–specific basic helix loop helix 1 (SOHLH1; accession number: NM_001101677.1),^20^ and 70–associated protein with cilia and flagella–associated protein 70 (CFAP70; accession number: NM_145170.3).^21^ Serum Ab markers were detected using purified GST‐fusion proteins as antigens. The five SEREX antigen markers identified were significantly higher in patients with ESCC compared to healthy donors (HDs). Similar results were obtained by receiver operating curve (ROC) analysis. Anti‐FIRΔexon2 antibodies (Abs) have been reported as a common candidate biomarker for ESCCs.^22^, ^23^, ^24^ The combined ROC analysis of candidate markers with clinically available tumor markers, such as anti‐p53 Abs, showed increased area under the ROC curve (AUC) values in the sera of patients with ESCC. Therefore, anti‐FIRΔexon2 Abs with anti‐p53 Abs or CEA improves the specificity and sensitivity to detect ESCCs.^25^

Therefore, the use of combinational Ab detection approaches could allow for the precise early detection of tumors.^26^ This study aims to investigate the significance of anti‐FIRΔexon2 Abs, and whether it increases the specificity and accuracy of CRC diagnoses with other clinically available tumor markers, such as anti‐p53 Abs, CEA, and CA19‐9, as reported in other cancer types.^26^, ^27^

## 2 MATERIALS AND METHODS

### 2.1 Clinical samples

The present study was conducted according to the Code of Ethics of the World Medical Association (the Declaration of Helsinki). The sera of patients with CRC (*n* = 90) were obtained from the Department of Frontier Surgery (Chiba University Hospital, Chiba, Japan). Blood samples were obtained from consecutive patients from 2012 to 2015. All the patients were histologically confirmed, and those with cancer were pathologically diagnosed with CRC. All blood samples from cancer patients were taken before any treatment. Sera from HDs (*n* = 94) was obtained from the Higashi Funabashi Hospital (Funabashi, Japan) as a control. HD blood samples were obtained from consecutive patients who had undergone brain checkups between 2013 and 2014. According to the inclusion criteria for healthy controls, people with a medication history and lifestyle‐related diseases were excluded.^28^ Written informed consent was obtained from all participants before the study. Each serum sample was centrifuged at 2000 × *g* for 10 min, and the supernatant was stored at −80°C until further use. Repeated thawing and freezing of the samples were avoided. The extraction of clinical data was conducted by one reviewer and checked by a second reviewer.^29^ This study was approved by the Institutional Review Board (IRB) of Chiba University, Graduate School of Medicine, and the Higashi Funabashi Hospital. (Approved ethical review No. 814(386) and 677).

### 2.2 Screening by expression cloning

We performed recombinant DNA studies with permission from the Chiba University Graduate School of Medicine and per the rules of the Japanese government. We used a λZAP II phage cDNA library prepared from mRNA of T.Tn cells (esophageal cancer cell lines)^30^ and a commercially available human fetal testis cDNA library (Uni‐ZAP XR Premade Library; Stratagene, La Jolla, CA) to determine immunoreactive clones against serum IgG from patients with ESCC, as previously described.^31^ Then, *E. coli* XL1‐Blue MRF' was infected with λZAP II or Uni‐ZAP XR phage, and the expression of resident cDNA clones was induced after the infected bacteria onto NitroBind nitrocellulose membranes (Osmonics, Minnetonka, MN). Next, we pretreated the membranes with 10 mM isopropyl‐β‐D‐thiogalactoside (IPTG; Wako Pure Chemicals) for 30 min. The membranes with bacterial proteins were rinsed three times with 20 mM Tris–HCl (pH 7.5), 0.15 M NaCl, and 0.05% Tween‐20 (TBST), and nonspecific binding was blocked by incubation with 1% protease‐free bovine serum albumin (Nacalai Tesque, Inc.) in TBST for 1 h. The membranes were exposed to 1:2000 diluted sera of patients for 1 h. After three washes with TBST, the membranes were incubated for 1 h with alkaline phosphatase‐conjugated goat anti‐human IgG (Jackson ImmunoResearch Laboratories, West Grove, PA). We developed positive reactions using 100 mM Tris–HCl (pH 9.5) containing 100 mM NaCl, 5 mM MgCl_2_, 0.15 mg/mL 5‐bromo‐4‐chloro‐3‐indolylphosphate and 0.3 mg/mL nitro blue tetrazolium (Wako Pure Chemicals). Furthermore, positive clones were recloned twice until monoclonality was obtained, as previously described.^32^, ^33^

We converted monoclonal phage cDNA clones to pBluescript phagemids by in vivo excision using the ExAssist helper phage (Stratagene). Then, we obtained plasmid pBluescript‐containing cDNA from the *E*. *coli* SOLR strain after transformation by the phagemid. Next, we evaluated the sequences of cDNA inserts for homology with identified genes or proteins within the public sequence database (http://blast.ncbi.nlm.nih.gov/Blast.cgi).

### 2.3 Expression and purification of antigen proteins

We constructed the expression plasmids of glutathione‐S‐transferase (GST)‐fused proteins by recombining the cDNA sequences into pGEX‐4 T‐3 (GE Healthcare Life Sciences). Next, the inserted DNA fragments were ligated into pGEX‐4 T‐3 using Ligation Convenience Kits (Nippon Gene). We used ligation mixtures to transform ECOS™‐competent *E*. *coli* BL21 (DE3; Nippon Gene) and confirmed appropriate recombinants using DNA sequencing and protein expression analyses. The expression of the GST‐fusion proteins was then induced by treating the transformed *E*. *coli* with 0.1 mM IPTG for 3 h. We purified GST‐fused recombinant proteins by Glutathione‐Sepharose column chromatography per the manufacturer's instructions (GE Healthcare Life Sciences) and dialyzed against phosphate‐buffered saline, as previously described.^34^ Confirmation of purification has been described in previous studies.

### 2.4 AlphaLISA (amplified luminescence proximity homogeneous assay)

**FIGURE 1:**
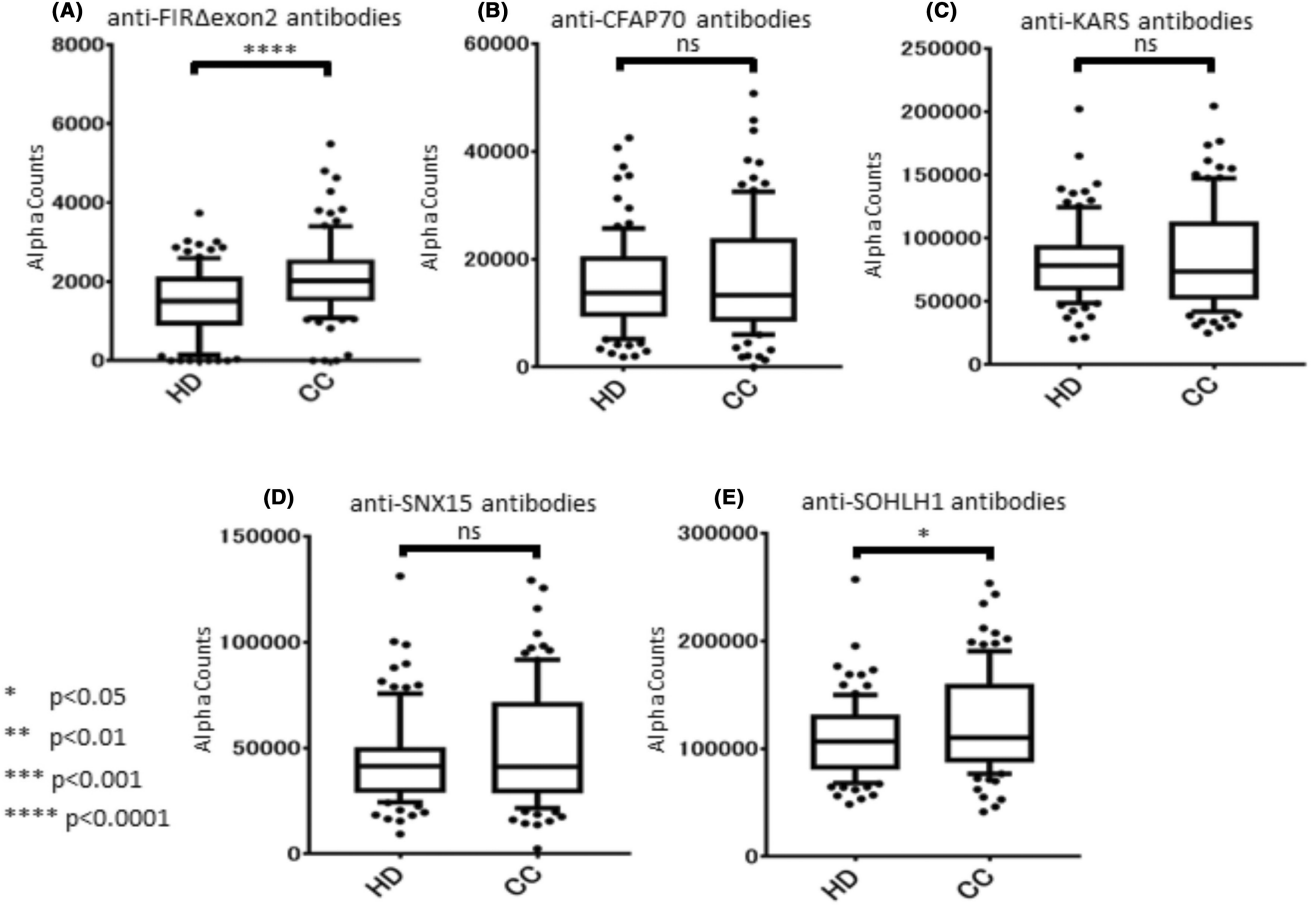
Comparison of antibody levels against serological identification of antigens by recombinant cDNA expression cloning (SEREX) and FIRΔexon2 antigens in patients with colorectal cancer (CRC). Antibody levels of antibodies (Abs) against FIRΔexon2, KARS, SNX15, SOHLH1, and CFAP70 Abs in healthy donors (HD) and patients with CRC measured by a homogeneous amplified luminescence proximity assay‐linked immunosorbent assay (AlphaLISA). Serum Ab levels measured by AlphaLISA are shown using a box–whisker plot. The box plots display the 10th, 20th, 50th, 80th, and 90th percentiles. Furthermore, the *p* values (calculated using the Mann–Whitney *U* test) are shown compared with the HD specimens. CC, Colorectal cancer; ns, not significant.

We performed AlphaLISA using 384‐well microtiter plates (white opaque OptiPlate™; PerkinElmer, Waltham, MA) containing 2.5 μL of 1:100 diluted sera and 2.5 μL of GST or GST‐fusion proteins (10 μg/mL) in AlphaLISA buffer (25 mM HEPES, pH 7.4, 0.1% casein, 0.5% Triton X‐100, 1 mg/mL dextran‐500 and 0.05% Proclin‐300). The reaction mixture was incubated for 6–8 h at room temperature. Then, anti‐human IgG‐conjugated acceptor beads (2.5 μL of 40 μg/mL) and glutathione‐conjugated donor beads (2.5 μL of 40 μg/mL) were added and incubated for 7–21 days at room temperature in the dark. The chemical emission was read on an EnSpire Alpha microplate reader (PerkinElmer), as previously described.^35^, ^36^ We calculated selective reactions by subtracting the Alpha values of GST control from the values of GST‐fusion proteins. Although AlphaLISA does not involve plate washing steps, it involves mixing antigens with Abs in sera, followed by the addition of donor and acceptor beads. For example, Figure 1 shows highly reproducible results, including distributions, *p*‐values, and positive rates, despite using different sets of sera from HDs and patients. The evaluation of suitable AlphaLISA conditions in this study revealed that incubation for 7–21 days is the best way to obtain specific antigen–antibody reaction, as well as decrease the noise background.^37^

Notably, as listed on their website, AlphaLISA is a registered trademark of PerkinElmer, Inc. (http://www.perkinelmer.com/lab‐solutions/resources/docs/GDE_ELISA‐to‐AlphaLISA.pdf).

### 2.5 Statistical analyses

In this study, all the statistical analyses were performed using GraphPad Prism 7 (GraphPad Software) and R‐3.5.1 statistical software. We used the Mann–Whitney *U* test to determine the significance of differences between groups. The predictive values of markers for CRC were examined by ROC analysis. Furthermore, we evaluated the Ab‐group‐specific *Z* scores to facilitate the comparison between all Ab groups. In addition, the *Z* score analysis was performed after normalization to the HD mean values:

*Z* score = (control mean) − (individual value)/(control standard deviation).

We performed the combined ROC analysis by adding each *Z score*, calculated using the formula above.^38^, ^39^ Furthermore, the AUCs were calculated and examined using the Delong or Bootstrap tests to compare the significant differences between the single or combined ROCs.^40^, ^41^, ^42^ The Delong test used the following formula:

$$D=\frac{V^{r}\left(\theta^{r}\right)-V^{s}\left(\theta^{s}\right)}{\sqrt{S^{r}+S^{s}}}$$

## 3 RESULTS

### 3.1 Higher level of anti‐FIRΔexon2 Abs was detected in the sera of patients with CRC

We analyzed serum autoantibody levels against FIRΔexon2, CFAP70, KARS, SNX15, or SOHLH1, by AlphaLISA in HD sera of HDs (Table S1) and patients with CRC. Anti‐FIRΔexon2 and anti‐SOHLH1 Ab levels were markedly higher in patients with CRC than HD (Figure 1). We determined the cutoff value as the average plus two standard deviations (SDs) of HDs (95% confidence interval). In addition, the percentages of antibody‐positive cases were as follows: anti‐FIRΔexon2 (11/90, 12%), anti‐CFAP70 (9/90, 9%), anti‐KARS (13/90, 14%), anti‐SNX15 (12/90, 13%), and anti‐SOHLH1 Abs (16/90, 17%), as shown in Table 1. Table 2 is the list of clinical features of CRC patients. In addition, Anti‐FIRΔexon2 and anti‐SOHLH1 Ab levels were not significantly associated with sex, age, clinical stages, CEA, CA19‐9, and anti‐p53 Abs level. Table 3 shows the diagnostic accuracy of Ab‐positive cases, a significant difference with HD.

**TABLE 1:** The percentage of antibodies‐positive cases on AlphaLISA assay.

	*n*	Anti‐FIRΔexon2 Abs (%)		*p*‐Value	Anti‐CFAP70 Abs (%)		*p*‐Value	Anti‐KARS Abs (%)		*p*‐Value	Anti‐SNX15 Abs (%)		*p*‐Value	Anti‐SOHLH1 Abs (%)		*p*‐Value
Healthy subject	94	1	(1)		5	(5)		3	(3)		5	(5)		2	(2)	
Colorectal cancer	90	11	(12)	<0.001	9	(9)	0.384	13	(14)	0.567	12	(13)	0.153	16	(17)	0.01

*Note*: *p‐*values were calcuated by the Mann–Whitney *U* test.

**TABLE 2:** List of clinical features of patients with CRC.

Colorectal Cancer		Anti‐FIRΔexon2 Abs (positive rate %)	*p*‐Value	Anti‐SOHLH1 Abs (positive rate %)	*p*‐Value
Sex	Male (54)	6 (11)	0.728	9 (17)	0.760
	Female (36)	5 (14)		5 (14)	
Age	≤70 years (45)	4 (9)	0.393	6 (13)	0.619
	>70 years (45)	7 (15)		8 (18)	
Dukes	A, B (49)	6 (12)	0.974	6 (12)	0.395
	C, D (40)	5 (13)		8 (20)	
	N.D. (1)	0 (0)		0 (0)	
CEA	Positive (36)	4 (11)	0.816	7 (19)	0.480
	Negative (54)	7 (13)		7 (13)	
CA19‐9	Positive (25)	5 (20)	0.226	6 (24)	0.251
	Negative (65)	6 (9)		8 (12)	
Anti‐p53 Abs	Positive (15)	1 (7)	0.508	3 (20)	0.675
	Negative (74)	10 (14)		11 (15)	
	N.D. (1)	0 (0)		0 (0)	

*Note*: Pearson's chi‐square test.

**TABLE 3:** Summary estimates of the diagnostic accuracy to predict patients with CRC.

	Sn (95% CI)%	Sp (95% CI)%	PPV (95% CI)%	NPV (95% CI)%	LR+ %	DOR (95% CI)
Anti‐FIRΔexon2 Abs	12.2 (7.0–20.6)	98.9 (94.2–100)	91.7 (64.6–99.6)	54.1 (46.6–61.4)	11.5	13.0 (2.2–141.1)
Anti‐SOHLH1 Abs	15.6 (9.5–24.4)	97.9 (92.6–99.6)	87.5 (64.0–97.8)	54.8 (47.2–62.1)	7.3	8.5 (2.1–38.2)

*Note*: Sn, Sp ets, Wilson‐Brown test; DOR, Baptista‐Pike test.

Abbreviations: DOR, diagnostic odds ratio; LR+, likelihood ratio of a positive test result; NPV, negative predictive value; Sn, sensitivity; Sp, specificity; PPV, positive predictive value.

### 3.2 Anti‐FIRΔexon2 Ab is the novel tumor marker candidate for patients with CRC

**FIGURE 2:**
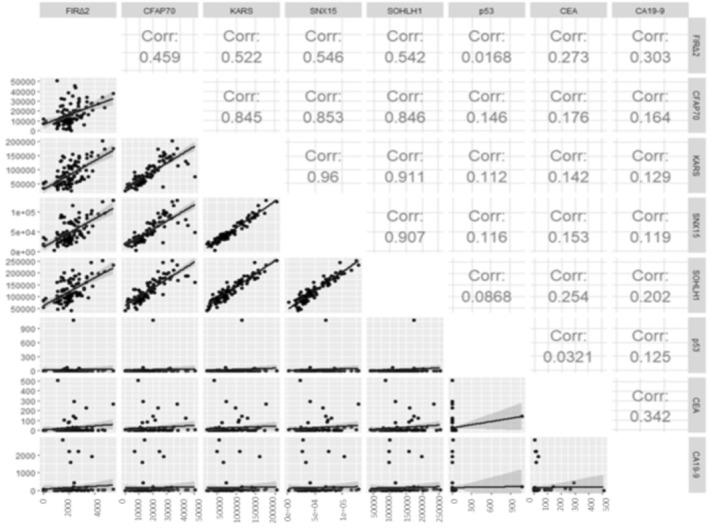
The correlation coefficient between candidate markers and clinically used tumor markers. The correlation was assessed using the Spearman rank correlation coefficient. Lower triangular matrix, pairwise scatter plots between variables; upper triangular matrix, Spearman rank correlation coefficients among each paired measurement. CFAP70, Anti‐CFAP70 Abs; FIRΔ2, Anti‐FIRΔexon2 Abs; KARS, Anti‐KARS Abs; SNX15, Anti‐SNX15 Abs; p53, Anti‐p53 Abs; SOHLH1, Anti‐SOHLH1 Abs.

Using Spearman's rank correlation analysis, we explored the existence of a correlation between anti‐FIRΔexon2, anti‐CFAP70, anti‐KARS, anti‐SNX15, or anti‐SOHLH1 Abs and clinically used tumor markers. The correlation coefficient between anti‐FIRΔexon2 Abs and clinically used tumor markers was not significant (Figure 2). Anti‐CFAP70 Abs were positively correlated with anti‐KARAS, anti‐SNX15, and anti‐SOHLH1 Abs but not with anti‐FIRΔexon2 Abs (Figure 2).

Results of the Venn diagram analysis for differentially detected markers identified in CRC patients. These were used to display the proportions of antibody‐positive cases shared among tumor markers. Therefore, anti‐FIRΔexon2 and anti‐SOHLH1 Abs were relatively independent of the CEA, CA19‐9, and anti‐p53 Abs (Figure S1). Specifically, the combination of clinically available tumor markers, CEA and CA19‐9, with anti‐FIRΔexon2 Abs as a novel biomarker candidate could improve the diagnostic efficiency and support the early detection of CRC.

### 3.3 Anti‐FIRΔexon2 Abs increased the AUC of CEA, CA19‐9, or anti‐p53 Abs in the ROC analysis

**FIGURE 3:**
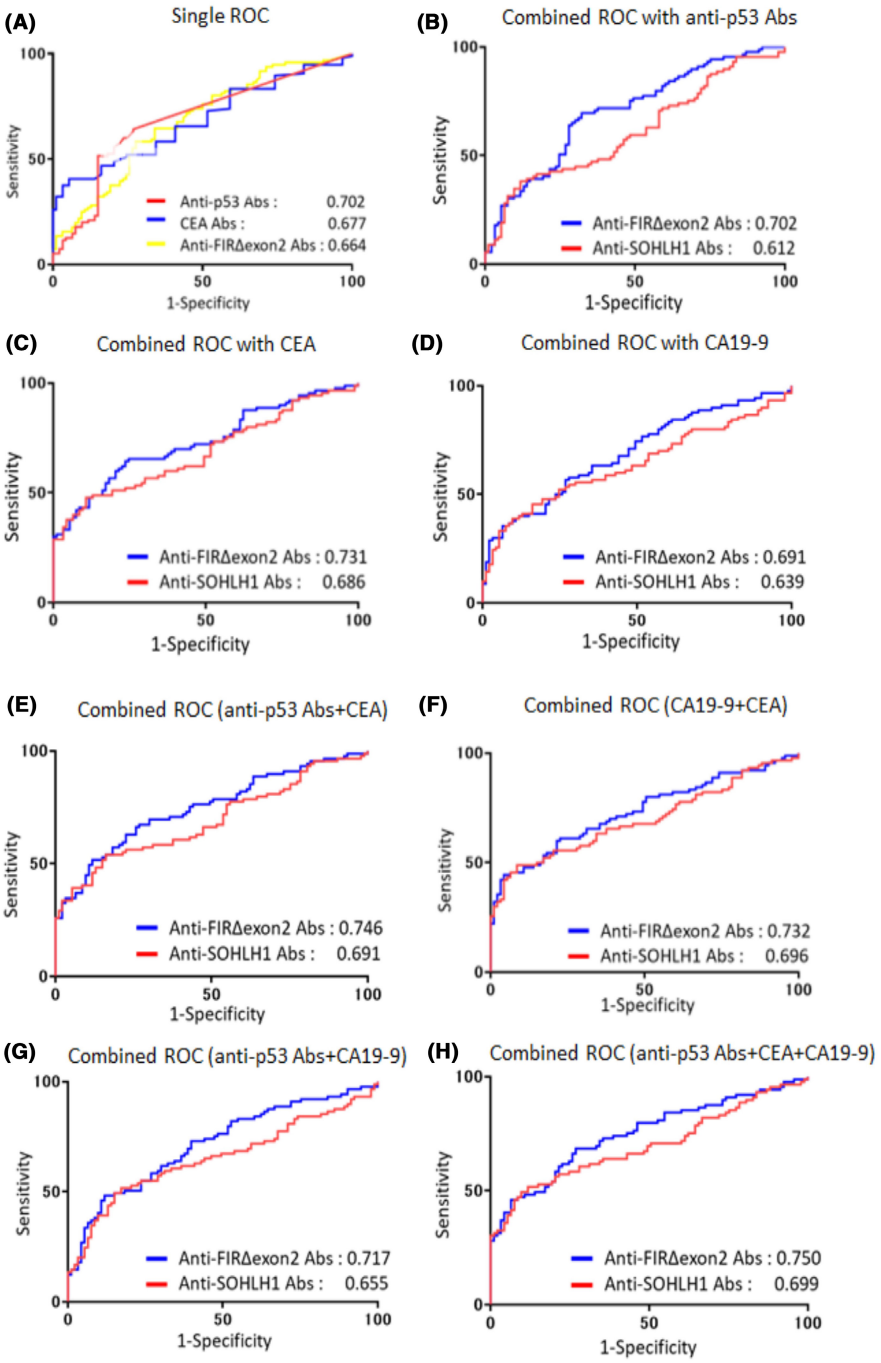
Comparison of the values of the area under the ROC curve (AUC) in patients with CRC. The overall diagnostic efficiency of seven markers was evaluated by comparing the receiver operating curve (ROC). The ROC curves were generated, and the AUC values were calculated using GraphPad Prism 7. (A) ROC analysis for individual candidate markers and CEA, CA19‐9, and anti‐p53 Abs markers. The values are shown in descending order of AUC. Shown are the top Ab lists. (B–H) ROC analysis for the combination of candidate markers and CEA, CA19‐9, and anti‐p53 Abs markers. ROC analysis of detected candidate markers and two clinically used tumor markers (CEA, CA19‐9, and anti‐p53 Abs) were created according to *Z* score data normalized to the standard deviation (SD) of the quantified Alpha count data of 90 patients with CRC and 94 HDs. The values are shown in descending order of AUC. Shown are the top Ab lists.

**FIGURE 4:**
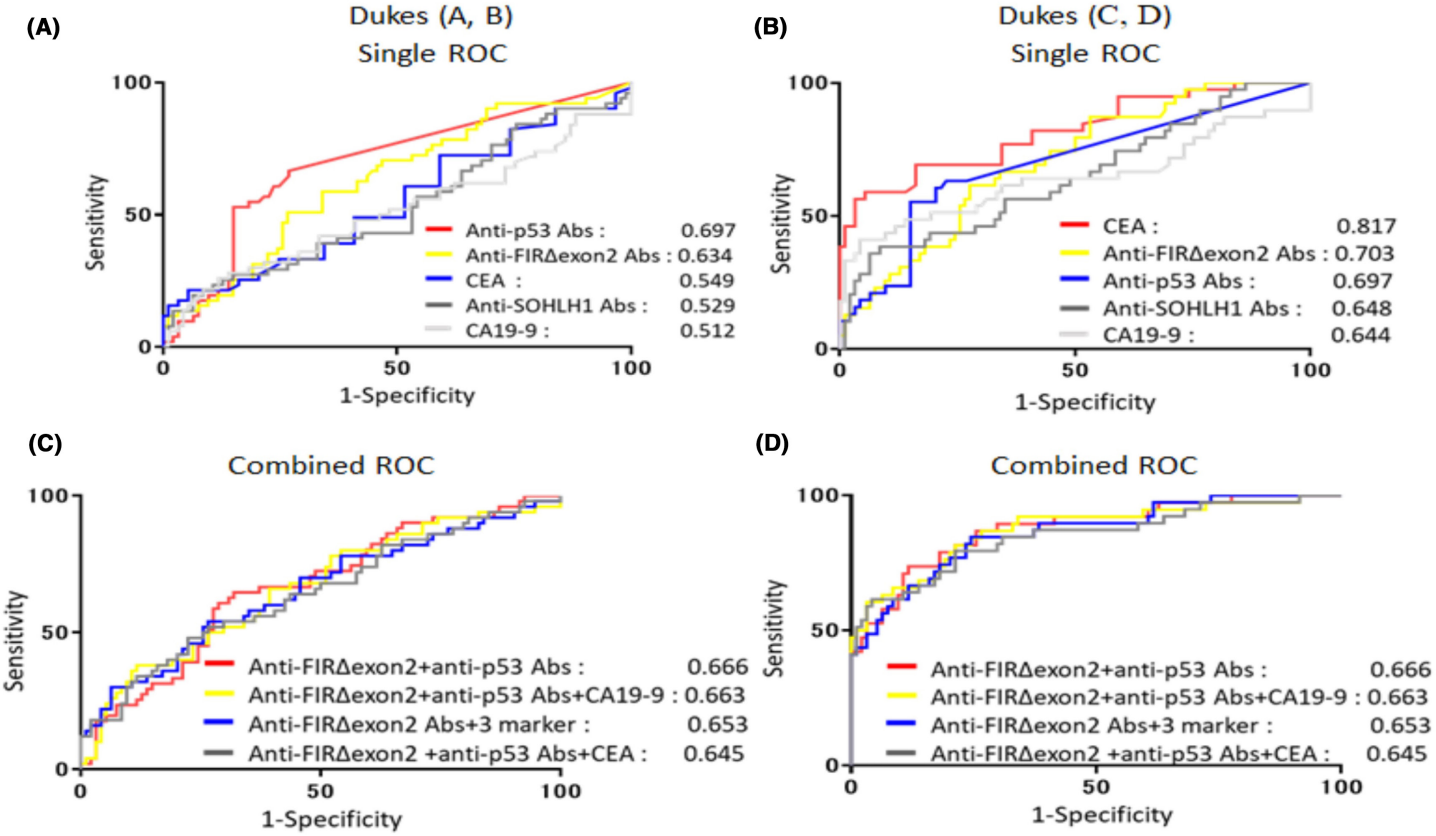
Receiver operating curve (ROC) analysis depicting the diagnostic efficiency of anti‐FIRΔexon2 Abs in combination with CEA, CA19‐9, and anti‐p53 Abs markers for early‐ or advanced‐stage colorectal cancer. The ROC analysis of detected candidate markers and two clinically used tumor markers (CEA, CA19‐9, and anti‐p53 Abs) was created according to the *Z score* data normalized to the standard deviation (SD) of the quantified Alpha count data of patients with CRC and HDs. (A) AUC values of candidate markers for early‐stage cancer. (B) The AUC values of candidate markers for advanced‐stage cancer. (C) ROC analysis that depicts the diagnostic efficiency of anti‐FIRΔexon2 Abs in combination with CEA, CA19‐9, and anti‐p53 Abs markers for early‐stage cancer. (D) The ROC analysis depicts the diagnostic efficiency of anti‐FIRΔexon2 Abs in combination with CEA, CA19‐9, and anti‐p53 Abs markers for advanced‐stage cancer.

We performed ROC analysis to evaluate the ability of candidate tumor markers—anti‐FIRΔexon2 and anti‐SOHLH1 Abs, to detect patients with CRC. The AUC value of anti‐p53 Abs (0.702) was the highest among those of anti‐SOHLH1 Abs (0.581), CA19‐9 (0.570), anti‐FIRΔexon2 Abs (0.664), and CEA (0.677) in patients with CRC (Figure 3A). To improve diagnostic efficiency, a combined analysis of detected candidate markers and existing tumor markers was considered. The AUC of anti‐FIRΔexon2 Abs + CEA (0.731) was higher than that of CEA (0.677) or anti‐SOHLH1 Abs + CEA (0.686) in patients with CRC when combined with clinically available tumor markers, (Figure 3C). Similarly, the AUC values combined with CA19‐9 or anti‐p53 Abs in patients with CRC were indicated (Figure 3B,D). Furthermore, when combined with both anti‐p53 Abs and CEA, the AUC of anti‐FIRΔexon2 Abs + CEA + anti‐p53 Abs (0.746) was higher than that of anti‐SOHLH1 Abs + CEA + anti‐p53 Abs (0.691), anti‐p53 Abs (0.702) or CEA (0.677) in patients with CRC (Figure 3E). Likewise, the AUC values combined with CA19‐9 + CEA or anti‐p53 Abs + CA19‐9 in patients with CRC were indicated (Figure 3F,G). Moreover, combined with anti‐p53 Abs, CEA, and CA19‐9, anti‐FIRΔexon2 Abs exhibited the highest AUC of 0.750 in sera of patients with CRC (Figure 3H). We examine the AUC according to the clinical stages, early stages (A and B), or advanced stages (C and D), in patients with CRC (Figure 4). The AUCs of the types of early clinical stage cancer types were as follows: anti‐p53 Abs (0.697), anti‐FIRΔexon2 Abs (0.634), CEA (0.549), anti‐SOHLH1 Abs (0.529), and CA19‐9 (0.512; Figure 4A). Moreover, for advanced clinical‐stage cancer types, the AUCs were as follows: CEA (0.817), anti‐FIRΔexon2 Abs (0.703), anti‐p53 Abs (0.697) anti‐SOHLH1 Abs (0.648), and CA19‐9 (0.644; Figure 4B). The combination result in early‐stage CRC, the AUC of anti‐FIRΔexon2 Abs with anti‐p53 Abs (0.666) was the highest (Figure 4C). In advanced‐stage CRC, the AUC of anti‐FIRΔexon2 Abs with CEA and anti‐p53 Abs (0.877) was higher than that of anti‐FIRΔexon2 Abs +3 marker (anti‐p53 Abs, CA19‐9 and CEA; 0.874) or anti‐FIRΔexon2 Abs + CEA (0.862) or anti‐FIRΔexon2 Abs + CEA + CA19‐9 (0.848; Figure 4D). Overall, the AUC of anti‐FIRΔexon2 Abs with CEA and anti‐p53 Abs (0.877) exhibited the highest AUC at the clinical stages in patients with CRC.

### 3.4 AUC values increased in the combined ROC analysis compared to the individual ROC analysis

**FIGURE 5:**
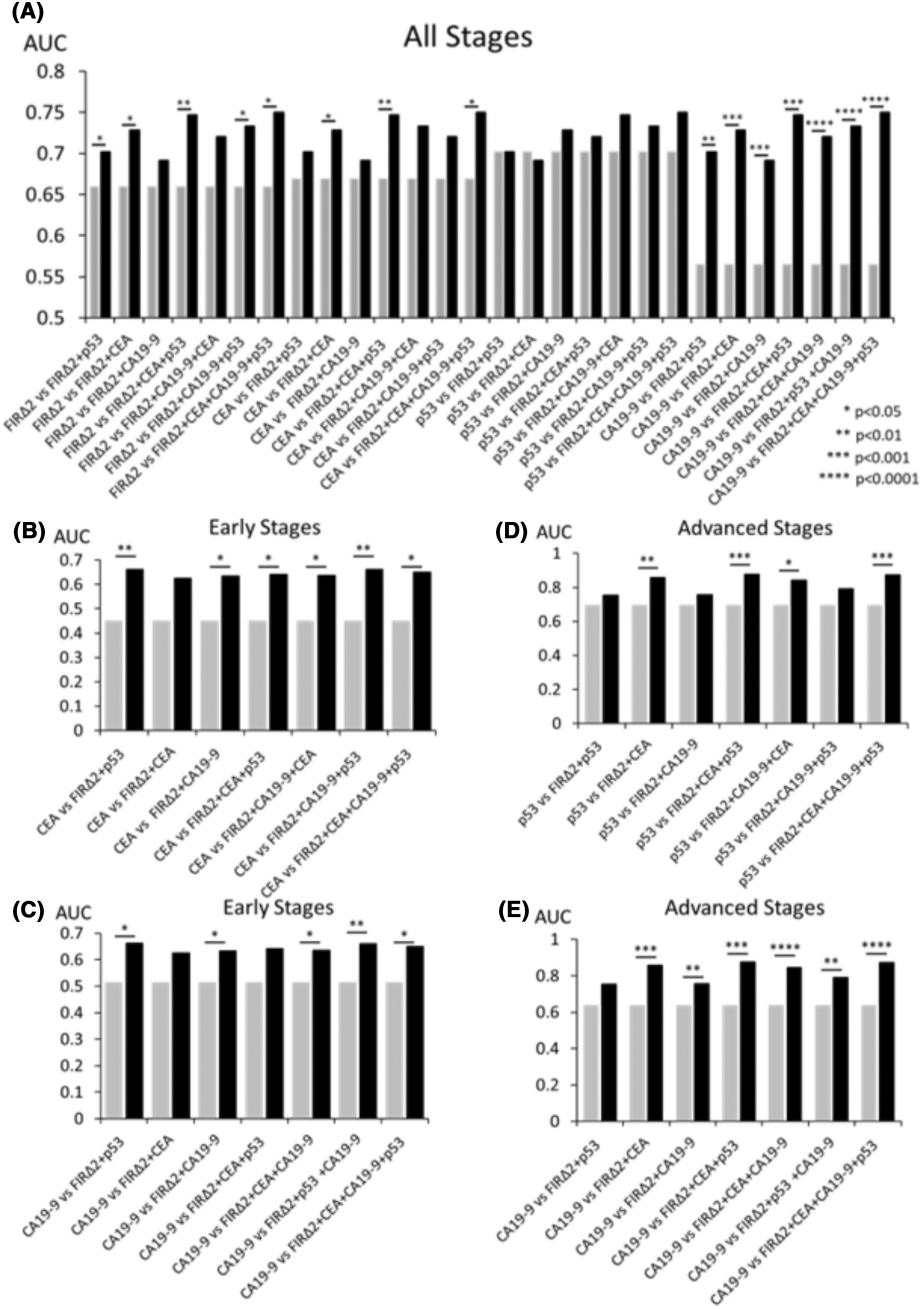
Comparison of AUC by colorectal cancer patients. The *Z* scores of the combined markers were calculated to facilitate the combined markers. The DeLong test examined the significance of AUCs among single or combined markers. (A) The significant difference obtained for cancer classified as all stages is shown. (B, C) The significant difference obtained for cancer classified as the early stage is shown. (D, E) The significant difference obtained for cancer classified as the advanced stage is shown. The *p*‐values were calculated using the Mann–Whitney *U* test. *p* < 0.05 was considered significant. FIRΔ2, Anti‐FIRΔexon2 Abs; p53, Anti‐p53 Abs; SOHLH1, Anti‐SOHLH1 Abs.

Ab‐specific *Z* scores were calculated to assess the significance of anti‐FIRΔexon2 Abs in CEA and CA19‐9 (Table S2) and clinical stages (Table S3). Furthermore, the significance of ROCs among single or combined markers has been assessed by comparing the AUC using Delong tests.^43^, ^44^ The levels of anti‐FIRΔexon2 Abs combined with clinically available tumor markers were significantly higher than those of anti‐FIRΔexon2 Abs (Figure 5A). The AUC of anti‐FIRΔexon2 Abs + CEA + anti‐p53 Abs was the highest among those compared with anti‐FIRΔexon2 Abs (*p =* 0.007). The levels of anti‐FIRΔexon2 Abs combined with clinically available tumor markers were significantly higher than CEA. The AUC of anti‐FIRΔexon2 Abs + CEA + anti‐p53 Abs was the highest among those compared with CEA (*p =* 0.012). No significant differences were found in AUC values between anti‐p53 Abs and levels of anti‐FIRΔexon2 Abs combined with clinically available tumor markers. The levels of anti‐FIRΔexon2 Abs combined with clinically available tumor markers were determined to be significantly higher compared with CA19‐9. The AUC of anti‐FIRΔexon2 Abs + CEA + CA19‐9 was the highest among those compared with CA19‐9 (*p* < 0.0001). The significant difference obtained for cancer classified as the early or advanced stage is shown in Figure 5 (B–E). In early‐stage CRC cancers, the *p*‐values of anti‐p53 Abs with anti‐FIRΔexon2 Abs (*p* < 0.01) were higher than those of CEA (Figure 5B). Similarly, the *p*‐values of CA19‐9 + anti‐p53 Abs with anti‐FIRΔexon2 Abs (*p* < 0.01) was higher than of CEA. Furthermore, in early‐stage CRC cancers, the *p*‐values of CA19‐9 + anti‐p53 Abs with anti‐FIRΔexon2 Abs (*p* < 0.01) were higher than those of CA19‐9 (Figure 5C). These results indicate that the levels of anti‐FIRΔexon2 Abs combined with clinically available tumor markers are useful in predicting the efficiency of early diagnosis. In advanced‐stage CRC cancers, the *p*‐values of CEA + anti‐p53 Abs with anti‐FIRΔexon2 Abs (*p* < 0.001) were higher than those of anti‐p53 Abs (Figure 5D). Similarly, the *p*‐values of 3 markers with anti‐FIRΔexon2 Abs (*p* < 0.001) was higher than those of anti‐p53 Abs. Furthermore, in advanced‐stage cancers, *p*‐values of CA19‐9 + CEA with anti‐FIRΔexon2 Abs (*p* < 0.0001) were higher than those of CA19‐9 (Figure 5E). Similarly, *p*‐values of 3 markers with anti‐FIRΔexon2 Abs (*p* < 0.0001) was higher than those of CA19‐9. These results indicate that the levels of anti‐FIRΔexon2 Abs combined with clinically available tumor markers are useful in increasing diagnosis efficiency.

## 4 DISCUSSION

This study reported that anti‐FIRΔexon2 Abs in sera is a novel diagnostic candidate marker and expects a better diagnosis for patients with CRC identified by SEREX screening and AlphaLISA.^25^ Furthermore, we have revealed that the combinational antibody detection approach could enable precise tumor diagnosis. Therefore, we examined and proposed some candidate markers for the early diagnosis of CRC. Compared with age‐matched HDs, anti‐FIRΔexon2 and anti‐SOHLH Abs in the sera of patients with CRC were markedly higher (Table 1; Figure 1). Furthermore, no correlation was observed between anti‐FIRΔexon2 Abs and the seven tumor markers (anti‐CFAP70, anti‐KARS, anti‐SNX15, anti‐SOHLH1 Abs, CEA, CA19‐9, and anti‐p53 Abs; Figure 2), indicating that anti‐FIRΔexon2 Abs is an independent marker for patients with CRC. The AUCs of anti‐FIRΔexon2 Abs were higher than those of the CA19‐9 and SEREX screening markers (Figure 3). Thus, anti‐FIRΔexon2 Abs is a potential novel biomarker candidate for patients with CRC.^24^, ^25^, ^27^ The combined ROC analysis of anti‐FIRΔexon2 Abs with clinically available tumor markers revealed higher AUC values in the sera of patients with CRC. Although the compatibility between markers differs depending on how they are combined, it became clear that increasing the number of markers' combinations improves diagnostic efficiency. Nevertheless, further prospective multi‐institutional studies will be required to determine the sensitivity and specificity of this combinational detection approach.

In our previous study, a high level of anti‐FIRΔexon2 Abs in the sera of patients with GC indicated better overall survival (OS).^45^ In contrast, in patients with CRC was not significant (Figure S2). The fact that CEA and CA19‐9 negative patients had better survival rates suggests the reliability of this data analysis. However, when combined with antibodies, the difference is no longer significant. This suggests that autoantibodies may be candidate biomarkers for immunoreactivity, which at present improves the probability of pre‐test diagnosis, but the clinical significance requires further investigation, including other organs. The OS of patients with CRC has been reported to be longer than that of patients with GC.^46^ The 5‐year CRC survival was between 50% and 60%^47^ and was higher in the initial stages (75%–90%) than in the advanced stages (< 15%).^48^ In the results of our study, CRC survival at 7–9 years is between 81.7% and is higher in the initial stages (92.5%) than in the advanced stages (67.5%). Regarding the prognosis, we could not confirm the additional effect of CEA, CA19‐9, and anti‐p53 Abs in combination with anti‐FIRΔexon2 Abs. Therefore, further longitudinal studies will be required to determine the better OS of patients with CRC of this anti‐FIRΔexon2 Abs.

In general, the levels of anti‐FIRΔexon2 Abs combined with clinically available tumor markers were determined to be significantly higher compared with CEA, CA19‐9. Furthermore, in early‐stage CRC cancers, the levels of anti‐FIRΔexon2 Abs combined with clinically available tumor markers were higher than those of CEA, CA19‐9. In advanced‐stage cancers, the levels of anti‐FIRΔexon2 Abs combined with clinically available tumor markers were higher than those of anti‐p53 Abs, CA19‐9. Therefore, the use of serum protein biomarker candidates holds promise for the development of inexpensive, noninvasive, and inexpensive tests for the detection of CRC. However, CEA is not recommended for use as a screening test for colorectal cancer.^49^ In addition, the data are insufficient to recommend CA 19–9 for screening, diagnosis, staging, surveillance, or monitoring of treatment of patients with colorectal cancer. Due to the highly heterogeneous nature of CRC, a single tumor marker is unlikely to become a standalone diagnostic test as the commonly insufficient sensitivity and/or specificity.^50^ Subsequently, using a combinational antibody detection approach of tumor markers for CRC diagnosis has the potential to be an effective approach.^51^

Furthermore, the analytical sensitivity of immunoassays is largely dependent on the avidity of the antibodies used in the assay and the lower detection limit of the label used.^52^ It should be noted that an appropriate quantity of chemibeads is essential for analytical performance. For instance, an excess amount of chemibeads might increase the probability of random collision between two types of nanoparticles, increasing the background signal and decreasing sensitivity. In contrast, an extremely low amount of chemibeads might decrease the intensity of chemiluminescence, thus reducing the sensitivity of the assay.^53^ In our previous study, we sought to identify anti‐FIRΔexon2 antibody markers for digestive organ cancers. Thus, the best cutoff values must be determined as with existing tumor markers. However, the anti‐FIRΔexon2 Abs titer for patients with CRC was 2047 Alpha counts, and 2237 Alpha counts for patients with GC. Anti‐FIRΔexon2 Abs titer for patients with ESCC was 2385 Alpha counts. However, the assay's sensitivity is not the only factor that determines titer differences in various types of digestive organ cancers. Driven by these results, we hypothesized that further assessments of some fundamental aspects of biomolecular assays were necessary.^54^ Therefore, in this study, we present a generic method for estimating the detection limit of biomolecular assays based on a step‐by‐step analysis of the assay procedure. The AlphaLISA detection limit could be improved if the binding affinity of the capture antibody with the target protein analyte could be increased accordingly.

The anti‐human IgG‐conjugated acceptor beads used in this study have six complementarity‐determining regions (CDRs). Antibody molecules specifically recognize target antigen molecules with these CDRs.^55^ Therefore, further optimization is important to improve the affinity of the antibody. Speed is required for clinical immunoassays, and neutralizing antibodies should react rapidly with their antigens. Furthermore, reducing the reaction time increases the operating rate of the autoanalyzers used for routine laboratory tests. The apparent association rate constant (kon) is a more important parameter than the equilibrium association constant: KA (or the equilibrium dissociation constant: KD) when the reaction time is limited, and antibodies with high‐kon values are preferable for practical applications. To obtain practical antibodies that allow for rapid and sensitive assays, an antibody with a high affinity against an antigen by their possessing a high‐kon value.^56^, ^57^ Based on these findings, the antigens we used were further refined. As a result, although the organ was different, the titer increased about four times. Furthermore, the detection rate also increased (Figure S3). Currently, implementing FIT screening would burden the colonoscopy reflex rate, colonoscopy complication numbers, facility costs, and patient distress by about 40%,^10^, ^58^, ^59^ while serial fecal occult blood tests are proven to reduce CRC mortality but suffer from significant false negative and false positive rates.^60^

In conclusion, the combination of anti‐FIRΔexon2 Abs with other clinically available tumor markers further improved the specificity and accuracy of a cancer diagnosis.

## AUTHOR CONTRIBUTIONS

Conception and design: Kazuyuki Matsushita, Hideaki Shimada, Fumio Nomura, Hisahiro Matsubara. Development of methodology: Takaki Hiwasa, Tyuji Hoshino. Provision of clinical samples: Takaki Hiwasa, Masayuki Kano, Hisahiro Matsubara. Acquisition of data: Sohei Kobayashi, Takaki Hiwasa, Kouichi Kitamura, Sho Hirano, and Mayuko Hashimoto. Analysis and interpretation of data: Sohei Kobayashi, Takaki Hiwasa, Masanori Seimiya, Kazuyuki Matsushita. Writing, review, and/or revision of the manuscript: All authors.

## FUNDING INFORMATION

This research received no external funding.

## CONFLICT OF INTEREST STATEMENT

The authors have no conflicts of interests to declare in this study.

## INFORMED CONSENT

Informed consent was obtained from the subjects and/or guardians.

## Supporting information


Figure S1

Figure S2

Figure S3

Table S1

Table S2

Table S3

## Data Availability

The data that support the findings of this study are available on request from the corresponding author. The data are not publicly available due to privacy or ethical restrictions.
