# Supplementary figures and images for: Combinational antibody detection approach increases the clinical validity of colorectal cancer screening
Source: J Clin Lab Anal. 2023 Nov 14;37(21-22):e24978. doi: 10.1002/jcla.24978 (PMC10749486; doi:10.1002/jcla.24978)

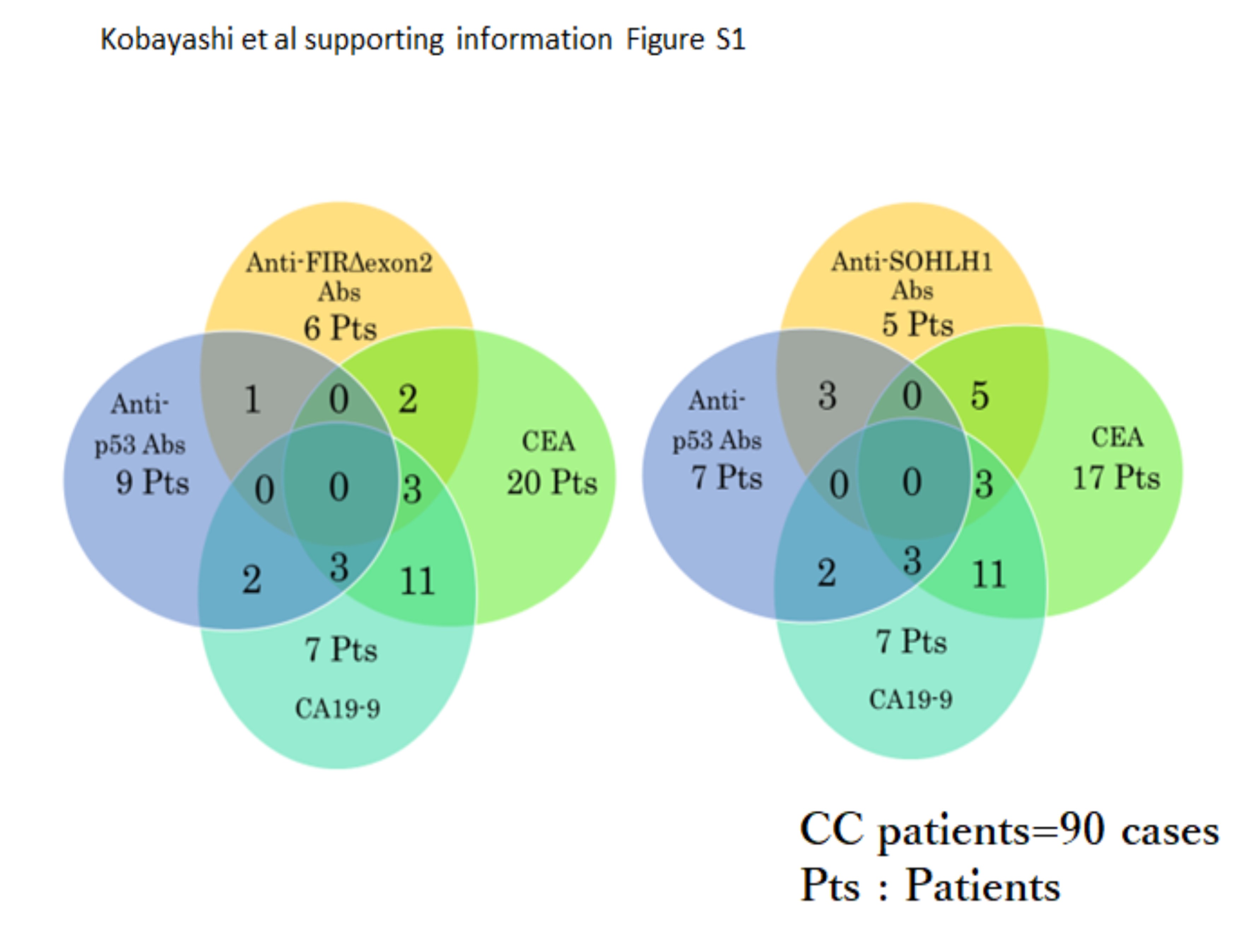

Supplement: Supplementary file 1 — Figure S1 [file JCLA-37-e24978-s006.jpg]

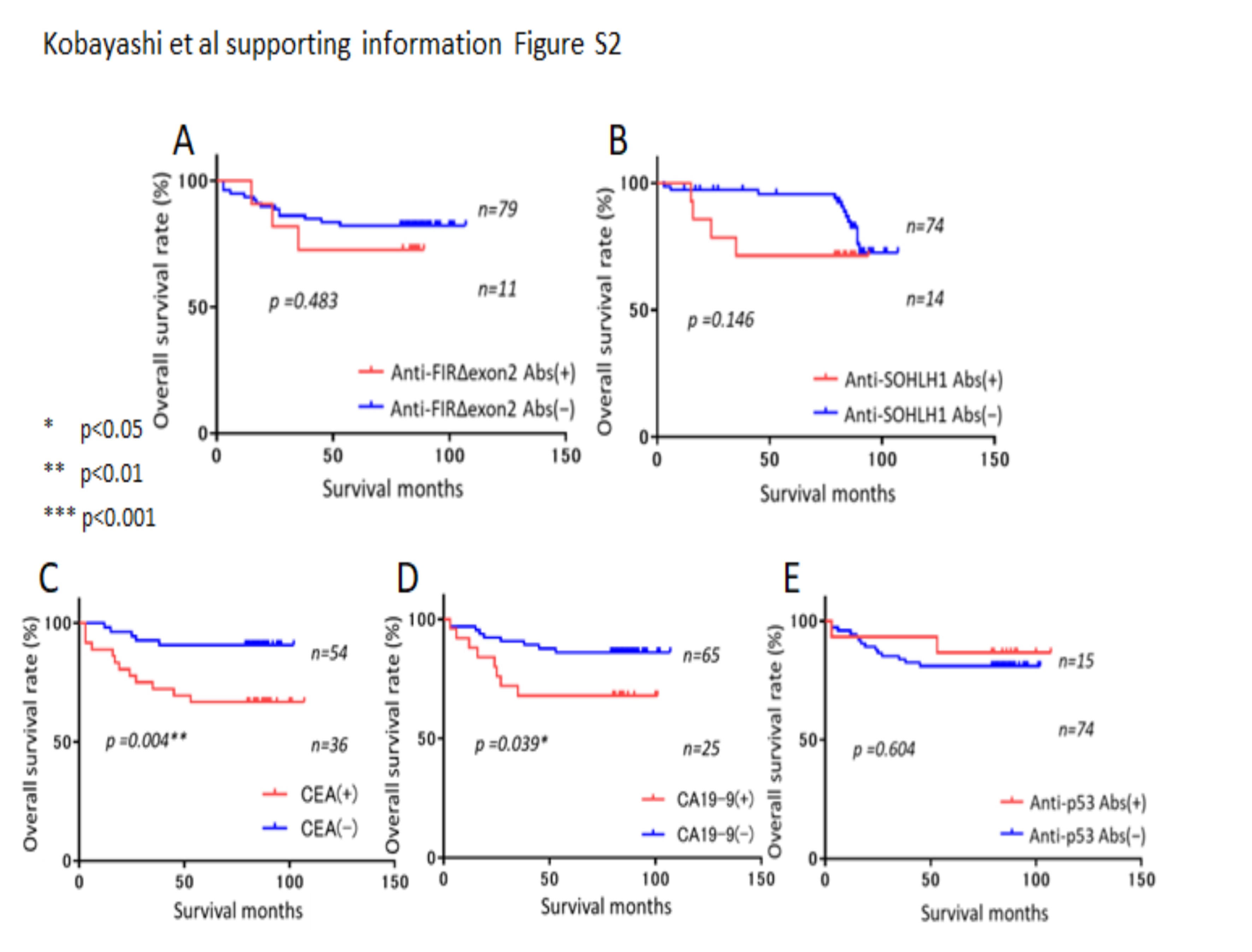

Supplement: Supplementary file 2 — Figure S2 [file JCLA-37-e24978-s005.jpg]

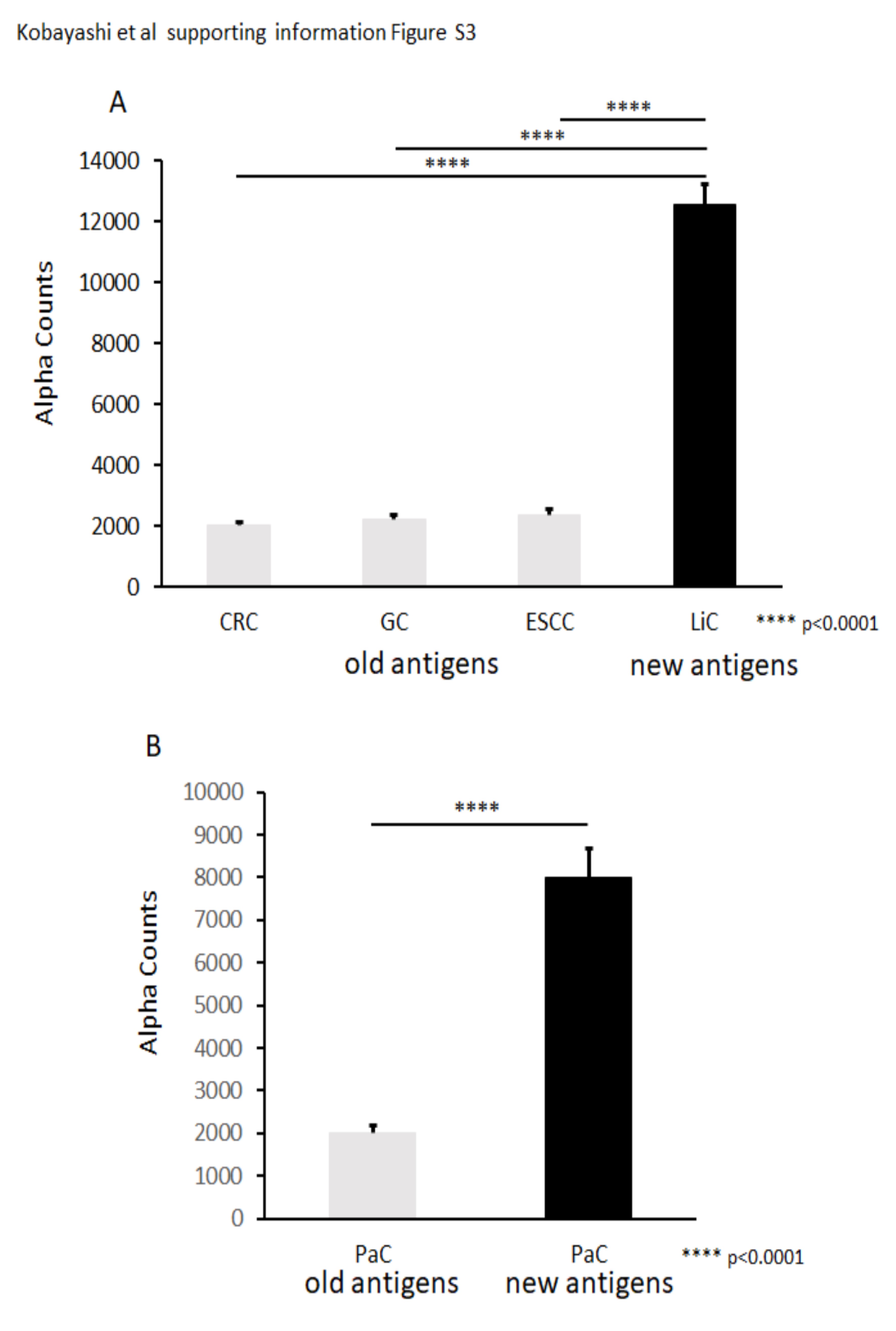

Supplement: Supplementary file 3 — Figure S3 [file JCLA-37-e24978-s003.jpg]
